# Factors Associated with Passive Sedentary Behavior among Community-Dwelling Older Women with and without Knee Osteoarthritis: The Otassha Study

**DOI:** 10.3390/ijerph192113765

**Published:** 2022-10-23

**Authors:** Naoki Deguchi, Narumi Kojima, Yosuke Osuka, Hiroyuki Sasai

**Affiliations:** 1 Research Team for Promoting Independence and Mental Health, Tokyo Metropolitan Institute of Gerontology, 35-2 Sakae, Tokyo 173-0015, Japan; 2 Department of Rehabilitation, Fukuoka Rehabilitation Hospital, 7-220 Nokata, Nishi, Fukuoka 819-8551, Japan

**Keywords:** physical activity, ecological model, sedentary time, interaction term, osteoarthritis

## Abstract

Passive sedentary behavior (SB) may lead to adverse health outcomes; however, it remains unclear whether the factors relevant to passive SB differ between older adults with and without knee osteoarthritis (KOA). This cross-sectional study examined factors associated with passive SB among 688 community-dwelling older women with (*n* = 128) and without (*n* = 560) KOA. Passive SB (min/day) was assessed using the Japanese-translated version of the Measure of Older Adults’ Sedentary Time questionnaire. Demographic, lifestyle, and psychosocial factors; pain; and physical performance were evaluated in multiple regression models with an interaction term concerning SB between the two groups. The mean (SD) total SB for those without and with KOA was 490.4 (200.9) min/day and 487.4 (185.8) min/day, respectively, and the majority of SB was passive, with no difference between groups. Passive SB was associated with an obese status and less time spent in group activities. However, no KOA-specific SB factors were identified. Therefore, effective interventions to promote social participation and weight loss, especially for individuals with obesity, may reduce passive SB, regardless of KOA. Longitudinal studies are warranted to identify causal relationships.

## 1. Introduction

Knee osteoarthritis (KOA) is a widespread chronic condition and one of the most common causes of musculoskeletal disability [1]. Sedentary behavior (SB) is defined as any activity that involves energy expenditure below 1.5 metabolic equivalents while in a seated or reclined posture [2]. As we age, the daily proportion of time spent in SB dramatically prolongs [3]. Recent evidence suggests that SB may be related to weight [4], depression [5], pain and disability [6], and physical function [7] in adults with KOA independent of moderate-to-vigorous physical activity (PA). In addition, sleep quality is negatively correlated with SB [8]. Obesity, a strong correlate of SB, is closely associated with KOA progression, and this association is stronger in women than in men [9]. Therefore, SB in older women with KOA requires special attention.

In a recent review [10], the SB consists of occupation, leisure, and mobility, and is further divided into two categories: passive SB (e.g., TV viewing, lying without sleeping, watching YouTube, and smartphone use), comprising mainly passive activities; and mentally active SB (e.g., reading, computer use, social media), involving greater amounts of mental activity. Studies of the two categories report that passive SB increases the risk of depression [10,11] and psychological distress [12]. In addition, social participation is associated with less passive SB time [13], yet mentally active SB is not associated with all these outcomes. These findings indicate that passive SB may have negative health effects. Thus, assessing the associations of passive SB with psychosocial, demographic, and lifestyle factors is also necessary. However, passive SB has only been investigated for healthy adults, not KOA. 

In healthy older adults, total SB is positively correlated with pain but not fatigue [14]; meanwhile, total SB in KOA is not correlated with pain but with fatigue [8]. These studies indicate that outcomes related to SB may differ by the presence or absence of KOA. Greater social participation leads to less time spent in passive SB, though not with total SB and mentally-active SB [13], and the presence or absence of a KOA is related to social participation [15]. This finding indicates that the association of pain and psychosocial factors with passive SB may differ depending on the presence or absence of KOA. However, there are no studies on KOA and passive SB, and the association between the two remains largely unknown. The socio–ecological model is a well-known framework of determinants of SB [16]. This model may be useful in identifying factors associated with SB.

We aimed to test whether factors associated with passive SB differ by the KOA status using a socio–ecological model among community-dwelling older women.

## 2. Materials and Methods

### 2.1. Study Design and Participants

**Figure 1:**
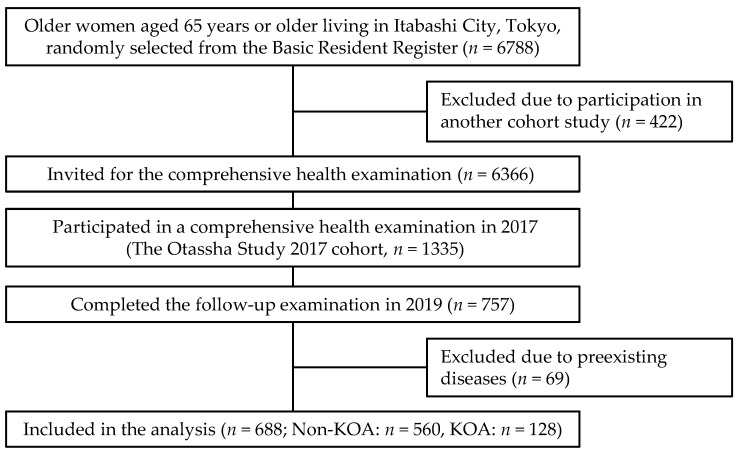
The flow of the study participants. KOA, knee osteoarthritis.

This cross-sectional study utilized data from a cohort study with a comprehensive health examination, “the Otassha study 2017 cohort” [17,18] conducted by the Tokyo Metropolitan Institute of Gerontology. Older women aged 65 years or older living in Itabashi, Tokyo, Japan were recruited using the Basic Resident Register (*n* = 6788) in 2017. After excluding 422 women who participated in another cohort study, we sent out invitation letters to 6366 candidates. A total of 1335 women participated in the health examination in 2017. Of them, 757 women (56.7%) completed the follow-up examination in 2019. Additionally, 69 women were excluded for Mini-Mental State Examination < 23 points (*n* = 6); brain disease (*n* = 22); stroke (*n* = 14); rheumatism and other collagen dis-eases (*n* = 24); dementia (*n* = 1); and Parkinson’s disease (*n* = 2); leaving 688 women for analysis (Figure 1).

This study used a self-reported physician-diagnosed KOA [19]. The participants were categorized into two groups based on their answers to the question: “Has a doctor ever told you that you have, or have ever had, KOA?”. Those who answered “No, never” were placed in the non-KOA group (*n* = 560), and those who answered “Yes” were placed in the KOA group (*n* = 128). All participants gave their oral and written informed consent before the study. The Institutional Review Board approved the study protocol, and the study was conducted in compliance with the Declaration of Helsinki ethical standards.

### 2.2. SB

The Measure of Older Adults’ Sedentary Time questionnaire was used to assess time spent SB during common activities [20,21]. The questionnaire was administered using a seven-item scale based on a one-week recall for domain-specific SB. Participants reported on activities performed during the last week while sitting or lying down (not including time spent in bed) and the total time spent: (1) watching television (TV), a video, or DVD; (2) using a computer; (3) reading; (4) socializing with friends/family; (5) traveling in a motor vehicle or on public transport; (6) doing hobbies; and (7) any other activities performed while sitting or lying down.

Time spent in SB was calculated by converting it to minutes per day. Total SB was calculated as the sum of the times spent performing each activity. Passive SB was calculated based on a previous study [12], including time spent watching TV/video/DVD, socializing, and other activities.

### 2.3. Socio–Ecological Model of Passive SB

Chastin et al. [22] identified factors associated with SB in older adults using a socio–ecological model through a systematic literature review, but those with diseases were excluded from their sample. Understanding socio–ecological factors that are most relevant to specific populations, and how these factors may interact in SB is necessary if SB is to be successfully targeted in interventions. Therefore, this study tests KOA-specific factors associated with passive SB, including demographic (age, obesity, and history of heart disease), lifestyle (PA and sleep disorders), psychosocial (subjective health status, fear of falling, depression symptoms, fatigue, and social participation), and pain and physical performance (knee pain, low back pain, five times sit-to-stand test [FTSST]).

#### 2.3.1. Demographic Factors

Age, obesity, and history of heart disease were recorded. Body mass index (BMI) was used to reflect the obesity status according to Japanese standards in which a BMI ≥ 25 is defined as obese [23]. Heart disease history was recorded as “no history” or “with history”.

#### 2.3.2. Lifestyle Factors

Exercise habits were determined by responses to questions regarding the frequency of participating in certain weekly activities [24]. For strolling or light exercise, participants indicated “yes” if they exercised every day, at least 5–6 days/week, or at least 2–4 days/week. They indicated “no” if they exercised less than or at least once per day. For regular exercise and sports, participants indicated “yes” or “no”. The criterion for the presence or absence of exercise habits was “not in the habit of exercising” if none of the responses was “yes”; otherwise, the criterion was “in the habit of exercising”.

Sleep disorder was measured using the Japanese version of the Pittsburgh Sleep Quality Index (PSQI-J), comprising 18 items [25,26], assessed using a four-point scale (0–3). A score of ≥6 is considered to indicate a sleep disorder.

#### 2.3.3. Psychosocial Factors

Subjective health was measured by asking: “In general, which of the following would you say best describes your health?”. For the analyses, the answer categories were: “very healthy” and “good health” (better self-rated health); or “not extremely healthy” and “not healthy” (poorer self-rated health).

Fear of falling was assessed by asking: “At this moment, are you afraid of falling?”. Answers were classified as “not at all”, “somewhat”, and “very much”. “Very much” was defined as a fear of falling [27].

Depressive symptoms were measured using the Japanese version of the Geriatric Depression Scale, with 15 items [28,29], recorded as “yes” or “no”. A score of ≥5 is considered to indicate a depressive state.

Fatigue was measured using the Chalder Fatigue Scale, a self-administered questionnaire with 14 items measured on a four-point Likert scale for the extent or frequency of symptoms [30,31]: 1 = very bad; 2 = not good; 3 = acceptable; 4 = very good.

Social participation was examined using question items from the National Health and Nutrition Survey in Japan [32]. The question related to this category was: “Do you participate in hobby groups and lessons?”. The responses were classified as: “not at all”, “somewhat”, or “very much”. Participants who responded “not at all” were defined as those without hobby groups. Moreover, the participants were asked: “Do you actively participate in neighborhood community associations (community), senior citizen clubs (senior club), or volunteer groups (volunteer)?”. Participants who responded “no” were defined as those without a group activity.

#### 2.3.4. Pain and Physical Performance

Participants who responded “yes” to the question: “Do you have knee pain?” were defined as having knee pain, the degree of which was evaluated as light, medium, or severe; responses “no” and “light” defined the group of no knee pain.

Low back pain was recorded as “no history” or “with history”. Participants with a history of disease were also asked about their current disease status and classified as “currently negative” or “currently positive”.

The FTSST was used to measure the time required to complete five consecutive sit-to-stand cycles as quickly as possible, timed using a stopwatch. The reliability and validity of the FTSST have been demonstrated in geriatric populations [33].

### 2.4. Statistical Analysis

Total and passive SB, demographic, lifestyle, psychosocial characteristics, and pain and physical performance were compared between the non-KOA and KOA groups using the Welch test for continuous variables and the chi-squared test for categorical variables. Multiple regression analyses were performed with passive SB as the dependent variable and demographic, lifestyle, psychosocial characteristics, pain, and physical performance as independent variables. The unstandardized regression coefficients and their 95% confidence intervals (CIs) were calculated. We included an interaction term between the group (with and without KOA) and independent variables for the regression analysis in the entire model. For these analyses, we tried to clarify the KOA-specific factors associated with SB using interaction terms for variables with *p* < 0.05 in the regression analysis.

The sample size was estimated considering eight participants per factor plus 50 participants for the multiple correlations and considering participants per factor plus 104 participants for the partial correlation [34]. As this study aimed to explore factors associated with passive SB, 14 factors were included, resulting in a minimal sample size of 162 older women. We created 20 datasets using multiple imputations by chained equations combined with Rubin’s rules to address missing data. Analyses were performed in R software (version 4.0.3 for Windows 64-bit, R Foundation for Statistical Computing, Vienna, Austria). All tests were two-sided and assessed at the 0.05 significance level.

## 3. Results

### 3.1. Participant Characteristics

Table 1 summarizes the participant characteristics. The women with KOA were older, had a greater BMI, higher rates of cardiac disease, slower FISST, more fall anxiety, and had more knee pain than those without KOA. The women with KOA also had higher scores on sleep disorder; however, there was no significant difference in the presence of sleep disorder between the two groups (*p* = 0.056).

### 3.2. Total and Passive SB

Table 2 summarizes the time spent in total and passive SB, and the percentage of passive SB time relative to total SB, demonstrating no significant differences between the two groups.

### 3.3. Factors Associated with Passive SB

Table 3 summarizes the factors associated with passive SB. Obesity was significantly associated with longer passive SB. Regular group activities were significantly associated with shorter passive SB. The interactions of obesity or group activity with KOA status did not reach statistical significance.

## 4. Discussion

This study investigated KOA-specific factors associated with passive SB among community-dwelling older women from multiple directions, including demographic, lifestyle, psychosocial, and pain/physical performance factors. The main finding is that no factors specific to KOA were significantly related to passive SB. Irrespective of the KOA status, obesity was positively associated with passive SB, and regular group activity was inversely associated with passive SB. Our findings suggest that the public health perspective for KOA should not be regardless of exercise habits but should also promote weight loss and social participation in individuals with obesity.

More consistent associations have been reported between TV viewing and the risk of obesity in older adults [35]. Furthermore, the risk of becoming obese was significantly positively associated with time spent watching TV, independent of meeting PA guidelines [36]. In this study, passive SB, including TV viewing and obesity were positively correlated, and 85.0% of the participants had a greater than twice-a-week exercise habit. These findings may indicate that it needs to consider the possibility that the impact on passive SB, such as TV viewing, may affect obesity, regardless of meeting the PA guidelines and exercise habits.

Previous studies have shown an association between KOA and social participation [15], and social participation and passive SB [13]; we expected that the passive SB of KOA would be affected by social participation. However, the association was not KOA-specific. A previous study demonstrated an inverse association between social activities that promote a cohesive neighborhood, TV viewing [37], and social participation in personal and community activities, showing that only the latter was associated with SB [38]. Similarly, we found that only community activities were associated with passive SB in the present study. Therefore, encouraging social community-based involvement may be necessary to reduce passive SB.

A previous study with Japanese adults [39] classified those with ≥8 h/day of total sedentary time as the high-sedentary group. In this study, regardless of KOA status, the proportion of total SB ≥ 8 h/day was ≥67.0%, and most of the sedentary time was spent with passive SB. These behaviors were comparable between older women with and without KOA. These findings suggest that effective interventions for health may require a decrease of total SB to target passive SB, regardless of the KOA status.

This study suggests that factors associated with passive SB may be less affected by KOA. However, several limitations should be considered when interpreting the current findings. First, the self-reporting SB scale used in our study has limited validity, potentially causing measurement error, and may result in underestimating the true associations. In contrast, objective measures of SB could not distinguish between different domains in which SB could take place. Future studies should continue to differentiate between domains, and objective measurements should be combined with subjective measures. Second, participants were classified as having KOA based on self-report. The radiographic definition presents higher estimates than self-reported OA definitions [40]. Lack of diagnostic information, including X-rays, also limits the widespread application of these results. Third, the study participants were women only. Factors associated with SB due to specific domain may vary by gender [41]. Thus, the results of this study may not be interpolated to older men. Lastly, the study data constitute one locality rather than a nationally representative population. Thus, the study has limited external validity.

## 5. Conclusions

The factors associated with passive SB did not differ by KOA status. To shorten passive SB, such as TV viewing time, promoting group activities and weight loss for obese individuals may be effective for older women, irrespective of KOA status. However, there remains a need for future longitudinal studies to investigate these factors.

## Figures and Tables

**Table 1:** Characteristics of the 688 older women.

	Overall (*n* = 688)	Non-KOA (*n* = 560)	KOA (*n* = 128)	*p* Value *
**Demographic factors**				
Age, mean (SD)	73.3 (4.2)	73.1 (4.2)	74.1 (4.2)	0.020
Body mass index, mean (SD)	22.6 (3.3)	22.3 (3.3)	24.0 (3.1)	<0.001
Obesity (body mass index ≥ 25), *n* (%)	141 (20.5)	95 (17.0)	46 (35.9)	<0.001
Heart disease, *n* (%)	81 (11.8)	58 (10.4)	23 (18.0)	0.024
**Lifestyle**				
Exercise habits, yes, *n* (%)	585 (85.0)	473 (84.5)	112 (87.5)	0.465
Walking, yes, *n* (%)	366 (53.2)	298 (53.2)	68 (53.1)	1.000
Light exercise, yes, *n* (%)	427 (62.1)	348 (62.1)	79 (61.7)	1.000
Regular exercise and sports, yes, *n* (%)	300 (43.6)	240 (42.9)	60 (46.9)	0.465
PSQI-J, points, mean (SD)	5.1 (3.1)	5.0 (3.1)	5.8 (3.1)	0.006
Sleep disorder (PSQI-J score ≥ 6), *n* (%)	254 (36.9)	197 (35.2)	57 (44.5)	0.056
**Psychosocial factors**				
Subjective health, yes, *n* (%)	633 (92.0)	517 (92.3)	116 (90.6)	0.647
Fear of falling, yes, *n* (%)	434 (63.1)	336 (60.0)	98 (76.6)	0.001
Depression symptoms, yes, *n* (%)	58 (8.4)	46 (8.2)	12 (9.4)	0.766
Chalder Fatigue Scale, mean (SD)	8.5 (6.1)	8.4 (6.2)	9.0 (6.0)	0.275
Hobby activities, yes, *n* (%)	258 (37.5)	211 (37.7)	47 (36.7)	0.919
Group exercise, yes, *n* (%)	254 (36.9)	204 (36.4)	50 (39.1)	0.649
**Pain and Physical performance**				
Knee pain, yes, *n* (%)	64 (9.3)	25 (4.5)	39 (30.5)	<0.001
Low back pain, yes, *n* (%)	253 (36.8)	197 (35.2)	56 (43.8)	0.087
FTSST, mean (SD)	9.4 (2.7)	9.2 (2.7)	10.1 (2.6)	0.001

SD, standard deviation; PSQ-J, The Japanese version of the Pittsburgh Sleep Quality Index; FTSST, five times sit-to-stand test. *: Non-KOA vs. KOA.

**Table 2:** Descriptive statistics of total and passive sedentary behavior.

	Overall (*n* = 688)	Non-KOA (*n* = 560)	KOA (*n* = 128)	*p* Value *
Total sedentary time, mean (SD)	489.8 (198.0)	490.4 (200.9)	487.4 (185.8)	0.877
Passive sedentary time, mean (SD)	330.8 (157.5)	331.7 (162.0)	326.6 (137.7)	0.743
Passive/Total sedentary time, %	67.5	67.6	67.0	

KOA, knee osteoarthritis; SD, standard deviation. *: Non-KOA vs. KOA.

**Table 3:** Factors associated with passive sedentary behavior and interactions with knee osteoarthritis (*n* = 688).

	Non-Interaction Term		Interaction Term with KOA	
	Est. (95% CI)	*p* Value	Est. (95% CI)	*p* Value
**Demographic factors**				
Age	–0.51 (–3.39, 2.37)	0.729		
Obesity (vs. No)	42.38 (11.99, 72.78)	0.006	–8.60 (–75.66, –58.46)	0.801
Heart disease (vs. No)	12.98 (–24.75, 50.70)	0.500		
**Lifestyle**				
Exercise habits (vs. ≥2 days/week)	–11.95 (–46.13, 22.24)	0.493		
Sleep disorder (vs. No)	–12.90 (–39.15, 13.35)	0.335		
**Psychosocial factors**				
Self-rated health (vs. healthy)	–32.33 (–78.78, 14.13)	0.172		
Fear of falling (vs. No)	–1.14 (–27.55, 25.26)	0.932		
Depressive symptoms (vs. No)	–19.54 (–65.45, 26.37)	0.404		
Fatigue (vs. No)	0.96 (–1.32, 3.25)	0.409		
Hobby activities (vs. well attend)	1.33 (–23.61, 26.27)	0.916		
Group exercise (vs. attend)	–30.26 (–55.62, –4.90)	0.019	8.71 (–54.35, 71.77)	0.786
**Pain and Physical performance**				
Knee pain (vs. No-Small)	14.04 (–31.48, 59.56)	0.545		
Low back pain (vs. No)	4.18 (–22.10, 30.46)	0.755		
FTSST	0.49 (–4.23, 5.20)	0.840		

CI, confidence intervals; KOA, knee osteoarthritis; FTSST, five times sit-to-stand test.

## Data Availability

The datasets generated during the current study are not publicly available due to ethical restrictions but are available from the corresponding author (H.S.) on reasonable request.
